# The application of a fully digital approach in the treatment of skeletal class III malocclusion: a preliminary study

**DOI:** 10.1186/s12903-023-02918-y

**Published:** 2023-04-24

**Authors:** Meng Li, Shunyao Shen, Zhiyang Zhao, Bo Wang, Hongbo Yu

**Affiliations:** 1 Department of Oral and Craniomaxillofacial Surgery, Shanghai Ninth People’s Hospital, College of Stomatology, Shanghai Jiao Tong University School of Medicine, Shanghai, China; 2 National Center for Stomatology and National Clinical Research Center for Oral Diseases, Shanghai, China; 3 Shanghai Key Laboratory of Stomatology and Shanghai Research Institute of Stomatology, Shanghai, China

**Keywords:** Digital technology, Orthognathic surgery, Invisalign, Skeletal malocclusion

## Abstract

**Background:**

Skeletal malocclusion patients have facial malformations and occlusal dysfunctions that require orthodontic-orthognathic joint treatment, while the combination treatment takes time and requires close communication between surgeons and orthodontists. Thus, improving the efficiency and effectiveness of the combination treatment is necessary, and it is still a challenge. Now, digital technology provides us with an excellent alternative. Despite the widespread use of digital technology in orthognathic surgery simulation and clear aligner orthodontic therapy, it has not been fully integrated into the combined orthognathic and orthodontic treatment process, and the components remain independent.

**Methods:**

A fully digital approach to seamlessly integrating various parts of the combined treatment through digital technology was investigated in this study in order to achieve an efficient transition. Five patients with skeletal Class III malocclusion were enrolled, and all made fully digital treatment plans at the beginning of actual implementation, which included the design of pre-surgical orthodontic, orthognathic surgery, and post-surgical orthodontic. Then, every aspect of the clinical operation was carried out in accordance with the fully digital routine. After the entire treatment process was completed, the skeleton and dentition discrepancy between virtual planning and the actual result was evaluated.

**Results:**

All participants completed the fully digital treatment process, and no complication was observed. The linear deviation of the skeletal anatomy was less than 1 mm, and the angular deviation was less than 1 degree. Except in one case in the lower dentition, the deviation of the virtual dental design from the real alignment was less than 2 mm. Furthermore, with one exception of maxillary anterior-posterior dimension, the linear deviations of the skeleton were not statistically significant. Therefore, the simulation accuracy of the fully digital approach was clinically acceptable.

**Conclusions:**

The digital treatment approach is clinically feasible and has achieved satisfactory results. The discrepancy between virtual design of the entire digital process and actual post-treatment situation was acceptable in clinic. A fully digital approach was proved effective in the treatment of skeletal Class III malocclusion, with which the efficient transition of treatment procedures was realized.

## Background

Different from simple malocclusion that can be corrected by orthodontics alone, dento-maxillofacial deformities need be treated with integrated surgical interventions. Preoperative and postoperative orthodontics are regularly needed for leveling and aligning the dentition to meet surgical requirements and obtain ideal final occlusion. Combined orthodontic-orthognathic treatment has a pivotal role in correcting maxillofacial deformities, which is a sequential process that necessitates sophisticated and practiced collaboration between surgical and orthodontic sections. However, due to the long-period features and multiple steps of joint treatment, communication between sections is prone to deviation, resulting in poor therapeutic effects and complications, or extended treatment duration [1]. At the same time, poor communication between departments impedes overall treatment plan implementation, increases the time and cost of follow-up, and even leads to medical disputes.

Digital technology provides us an optimal solution. Digital orthodontic-orthognathic joint treatment is becoming preferred for precise treatment of dento-maxillofacial deformities. Digital technology is primarily used in 3D cephalometric measurement, virtual surgical design, and 3D printing of guides and splints [2–6]. As a significant component of digital technology, virtual surgical planning (VSP) facilitates the effective combination and control of the whole treatment procedure. Similarly, based on digital technology, clear aligners have significant advantages in forecasting and visualizing tooth movement throughout the treatment process compared to conventional fixed appliance orthodontics with acceptable treatment outcomes [7–9]. In recent years, rising concerns about facial esthetic and periodontal health maintenance have accelerated the popularity of clear aligners in the correction of malocclusion [10, 11], such as the Invisalign system (unless specially stated, clear aligners mentioned below are equivalent to Invisalign orthodontic aligners). Inspiringly, clear aligners are used in orthodontic treatment followed by orthognathic surgery (OGS) and also achieve satisfied occlusion and postsurgical stability [12, 13]. However, clear aligners are not seamlessly integrated into the entire joint treatment process, leaving orthognathic and orthodontic procedures to be performed separately.

For the reasons above, this study was to explore the feasibility of a fully digital approach in the treatment of skeletal Class III malocclusion, to realize the seamless connection between orthognathic and orthodontic treatment, and to achieve greater efficiency and better therapeutic effects eventually.

## Methods

### Patients inclusion and analysis methods

Five patients were enrolled, selected from consecutive patients who underwent a fully digital approach in our hospital. The inclusion criteria were: (1) 18 years or older; (2) patients diagnosed as skeletal Class III malocclusion (ANB < 0); (3) facial symmetry: chin shifts less than 3 mm; (4) indication for two-jaw orthognathic surgery including Le Fort I and bilateral sagittal split ramus osteotomies; (5) mild to moderate crowding (6 mm or less per arch) and non-extraction orthodontic treatment was performed in both maxillary and mandibular dental arches; (6) receipt of clear aligner therapy. The exclusion criteria were: (1) craniofacial syndromes; (2) active periodontal disease; (3) history of orthodontic treatment.

Following enrollment, all participants had a personalized fully digital treatment plan and were required to adhere to the schedule strictly. The study was self-controlled. To investigate the clinical outcomes and simulation accuracy of a fully digital approach for skeletal Class III malocclusion, the immediate results after surgery were considered criteria for assessing the effectiveness of the surgical simulation. The dental alignment after postsurgical orthodontics was used to evaluate the effectiveness of the orthodontic simulation. All digital design and clinical management were conducted by the same orthognathic and orthodontic team.

### Fully digital procedure

#### Treatment plan

**Fig. 1:**
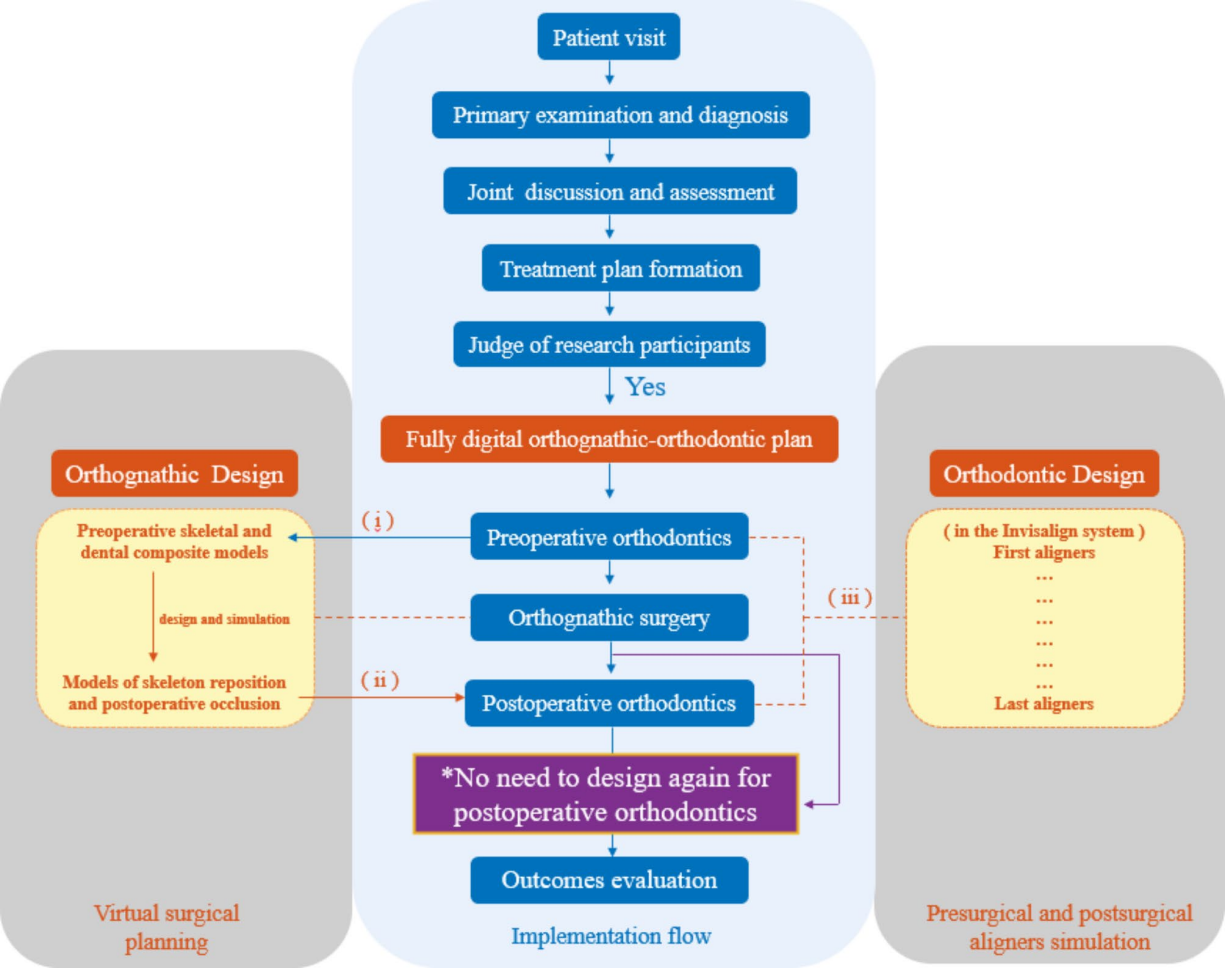
Fully digital routine in the treatment of skeletal Class III malocclusion The colorized dotted lines mean the simulation process of orthognathic surgery and orthodontics, which were accomplished at the beginning of treatment. The colorized solid arrows mean the interaction between OGS and orthodontics in the simulation phase and implementation process. (**i**) When preoperative orthodontics was finished, the last preoperative orthodontic model was used for dento-maxillofacial model reconstruction, based on which a virtual surgical plan was performed. (**ii**) The postoperative occlusion obtained from the virtual surgical design was in accordance with the postoperative dental alignment obtained from the Invisalign simulation prior to surgery. (**iii**) The whole orthodontic process was simulated initially with Invisalign at the beginning, containing preoperative and postoperative orthodontics. The preoperative phase was designed to meet the needs of OGS, and based on surgery simulation, immediate postoperative dentition can be attained early to design subsequent orthodontics. Thus, the entire procedure of orthodontics and orthognathic surgery is fully digital and seamless

Clinical data were collected at the beginning for all patients, such as CT, dental plaster casts and 3 dimensional photographs. Each CT had a pixel size of 0.45 mm x 0.45 mm, a slice interval of 1.25 mm, and a resolution of 512 × 512 × 231 (LightSpeed Ultra 16 spiral CT machine, GE Company, USA). Then, a joint orthognathic-orthodontic consultation was conducted to make a personalized treatment plan. Detailed procedures for preoperative orthodontic treatment, orthognathic surgery, and postoperative orthodontics were defined. With the help of multiple digital technologies, intensive communication and feedback were achieved throughout the entire procedure (Fig. 1).

#### Invisalign simulation and preoperative orthodontic treatment

The patient’s denture and occlusal relationship were obtained by iTero Element intraoral scan (Align Technologies, San Jose, CA, USA). Then, the 3D scanned files were imported into ClinCheck software (Align Technology, Santa Clara, CA, USA) for subsequent teeth movement design based on a digital dentition model. For skeletal Class III malocclusion, the primary purpose of preoperative orthodontics includes sufficient decompensation of both upper and lower anterior teeth, which directly determines the amount of sagittal skeletal correction during orthognathic surgery. With the help of ClinCheck software, the amount of lower anterior teeth torque and upper molar distalization could be precisely calculated to ensure that the anterior teeth were fully decompensated and adequately located in accordance with the inclination of the alveolus. In general, surgical intervention will change the occlusal relationship, which isolates pre- and post-orthodontic design. Benefit from the simulation function of the Invisalign system, the orthodontist can take occlusal changes caused by OGS into virtual planning consideration, thus integrating two parts of orthodontics to avoid separation by intermediate surgery, which means changes caused by surgery were firstly simulated in this period by fully digital approach. The whole process of orthodontic-orthognathic joint treatment was seamlessly designed and simulated with the Invisalign system, and then the digital models would be sent to the manufacturing factory to produce clear aligners. All participants were informed to wear clear aligners 20–22 h per day and change them sequentially every 10 days. The periodic orthodontic appointment was made every 2 months.

#### VSP and orthognathic surgery

When the preoperative orthodontic treatment finished, the next section of OGS was activated. The last digital dentition model of preoperative orthodontic treatment was combined with maxillofacial CT scan (LightSpeed Ultra 16 spiral CT machine, GE Company, USA) to reconstruct the dento-maxillofacial model using ProPlan CMF 3.0 software (Materialise, Leuven, Belgium). Based on this, a virtual surgical plan was developed, and the postoperative occlusal relationship was defined according to the Invisalign simulation, as mentioned in the previous step. Then guides and splints were 3D printed.

Orthognathic surgery, including Le Fort I osteotomy and bilateral sagittal split ramus osteotomy (BSSRO), was performed according to the surgical plan. The clear aligners were removed before the operation in order to avoid obstructions in the process of seating the intraoperative splint. Splints were used to reposition the segmented maxillary and mandibular bone sections, which were subsequently fixed in a new place with titanium plates and cortical bone screws. The final splint was fixed on the maxilla, and intermaxillary elastic distraction was applied to maintain the new occlusal relationship.

#### Postoperative orthodontic treatment

One month after surgery, the splint was removed, then postoperative orthodontics was activated, proceeding with the remained part of the orthodontic design.

### Accuracy evaluation

**Fig. 2:**
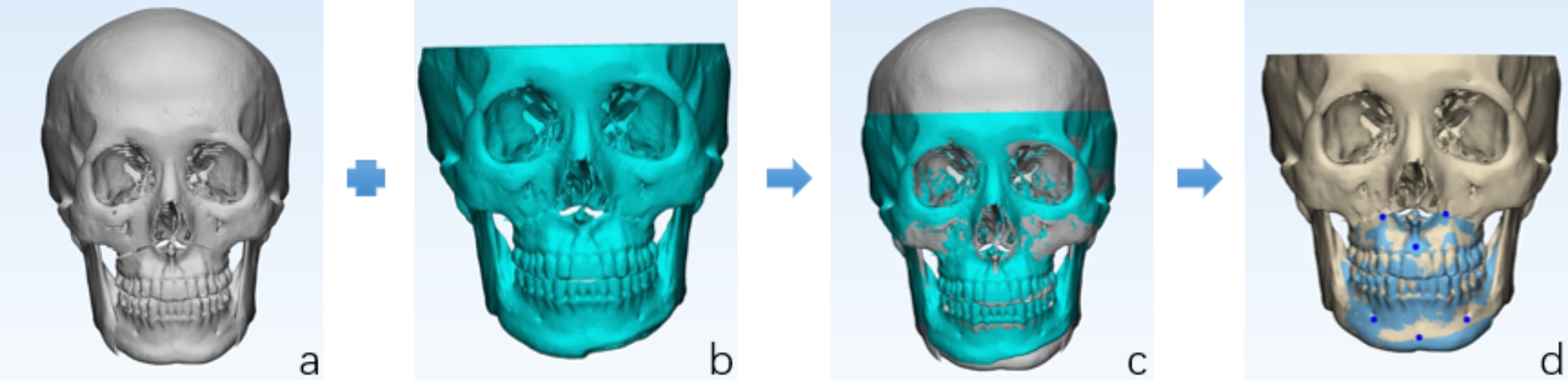
Image fusion process (**a**) model of digital simulation; (**b**) post-treatment CT reconstruction model; (**c**) superimposition of two models; (**d**) marking reference points on maxilla and mandible segment

The digital models used in this study encompass virtual surgical planning and Invisalign dentition simulation, while the post-treatment outcome involves skeletal anatomy and dental alignment. These were obtained through CT reconstruction and dental plaster casts after the treatment. To assess the deviation between the fully digital planning and the actual outcome, image fusion of two models was performed, which were obtained during the digital design stage and the post-treatment stage (Fig. 2a and b).

**Fig. 3:**
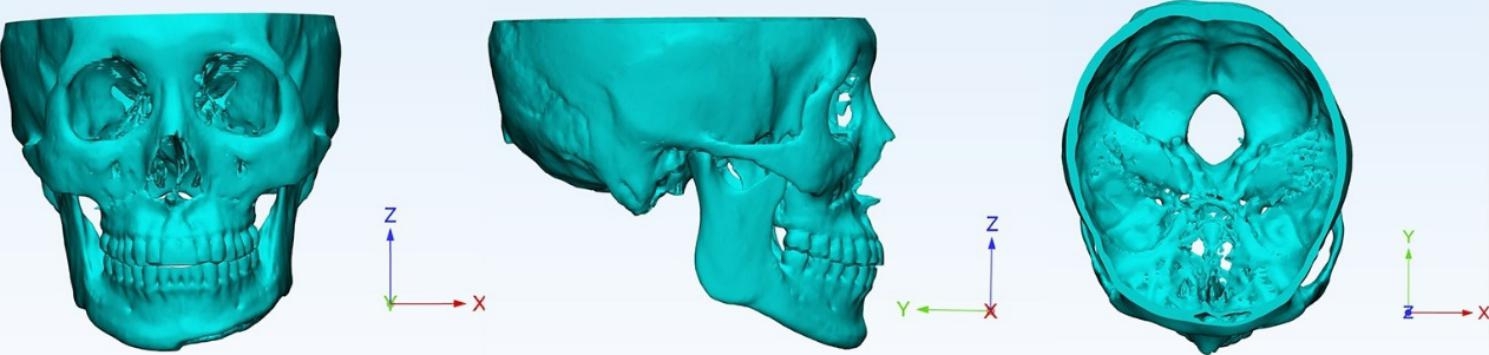
Spatial coordinate system

Firstly, CT scans reconstruction models were established with ProPlan software and subsequently registered to the simulation model using the surface best-fit method [14, 15]. The maxillary superimpositions were aligned with the midface, which was stable during the treatment period. As the mandibular was mobile during the surgery, it was superimposed by condylar head position (Fig. 2c). Following this, a spacial coordinate system was established as the original CT data in three axes, respectively x-axis representing mediolateral dimension, y-axis representing anteroposterior dimension, z axis representing superoinferior dimension (Fig. 3). To reflect the dimensional position of maxillary and mandible, three points were separately applied on each anatomy to generate centroid to calculate the linear difference and angular difference. These three points in space were indicative of the position and orientation of the anatomy [15]. All superimposition and reference point determination processes were performed using ProPlan software by the first authors, in the “Scan registration wizard” of the “Segment” module and the “Measure and Analysis” of the “CMF/Simulation” module, respectively.

For maxilla, bilateral greater palatine foramen and incisive foramen were used as reference points. Respectively, bilateral mental foramen and pogonion were used in the mandible. Then, three reference points were marked on the maxillary segment of the simulation model, cut by the Le Fort I osteotomy line, and the segment was then registered to the post-treatment CT reconstruction model using the surface best-fit method to obtain the digital design deviation, avoiding errors caused by manual marking of landmarks. In mandible, three reference points were placed on the segment of the mandibular body of simulation model that was cut off by the BSSRO osteotomy line, and the simulation accuracy of the mandibular position was evaluated in the same way (Fig. 2d). All coordinate values (x, y, z) of reference points were imported into MATLAB R2012b (The MathWorks Inc., Natick, MA, USA) to calculate simulation errors. The accuracy of skeletal anatomy simulation was evaluated for linear deviation and angular deviation, the posterior of which was calculated as rotational difference along x, y, and z, respectively defined as pitch, roll, and yaw. Furthermore, the linear deviation was an orthogonal difference along the x, y, and z axes.

**Fig. 4:**
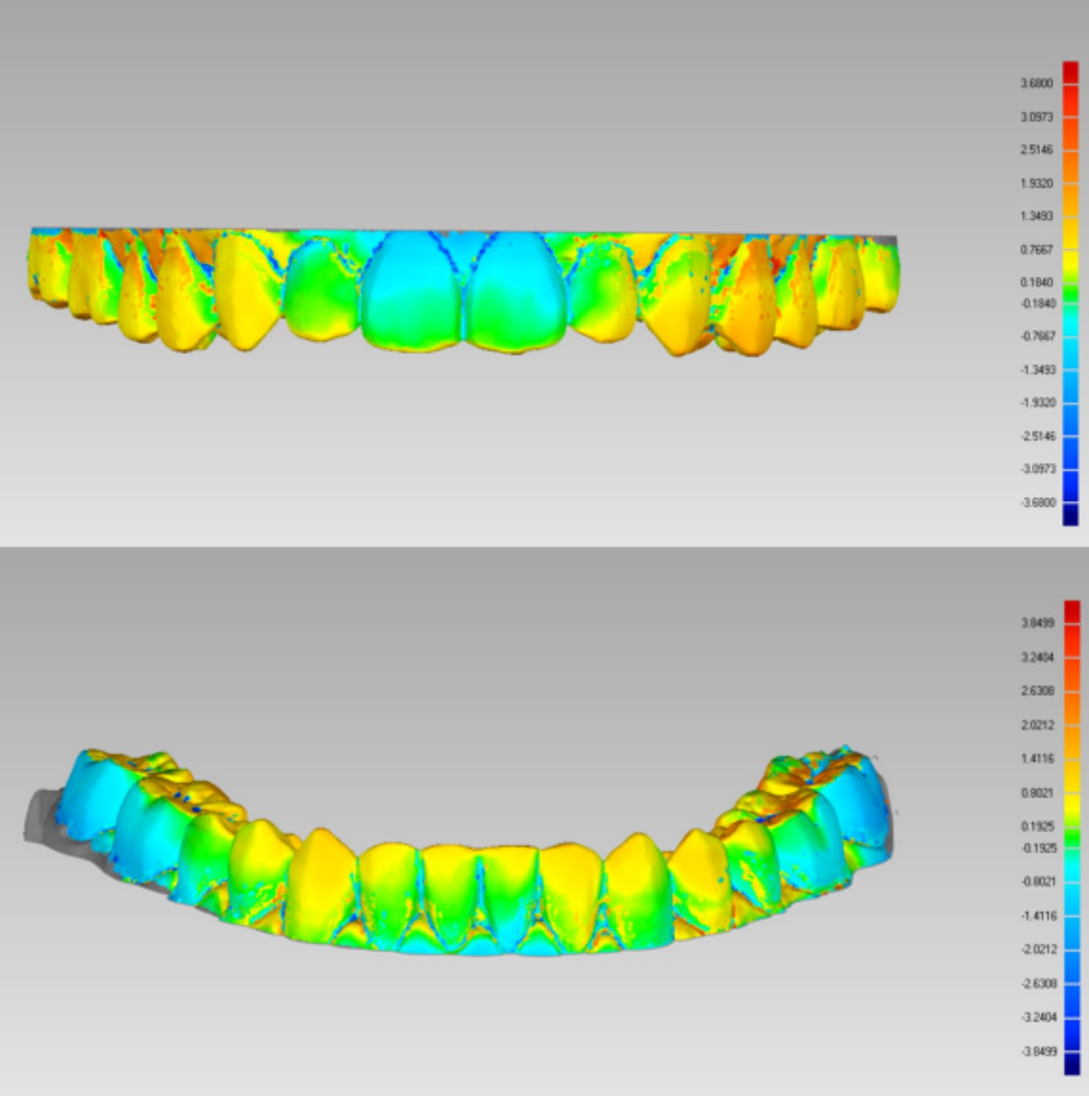
Discrepancy analysis of dental alignment between simulation and post-treatment actual dentition

The precision of the virtual design for maxilla and mandible focused more on the correct spatial position of the bone segments and did not care about the shape fit of the surgical design to the post-treatment bone segments, which could undergo alterations that significantly deviate the results. For the accuracy of the virtual design of the dentition, more focus should be on the fit of the virtual design to the actual dentition, as this is more evident for the occlusal alignment. Post-treatment dental plaster casts were obtained and scanned for digital models. The model of the last Invisalign design aligners was superimposed with the post-treatment model by a regional registration method [16]. This process was employed on Geomagic Studio 2013 software as 3D surface-to-surface matching by a least-mean-squared algorithm (Fig. 4).

### Statistical analysis

The descriptive statistics of deviations were calculated and normal distribution was tested. The linear and angular deviations were then imported into IBM SPSS Statistics version 25 (IBM Corp., Armonk, NY, USA). The Kolmogorove Smirnov and Shapiroe Wilk tests were used to test distribution normality. If the variables were normally distributed, paired t-test was performed. If not, Wilcoxon signed-rank test was carried out. A P value less than 0.05 was considered to be statistically significant different.

## Results

All participants got satisfying clinical outcomes under a fully digital approach and no complications were found. The linear and angular deviation of the maxilla and mandible were described in Table 1. The maxilla’s median linear errors in the x, y, and z dimensions were 0.17 mm, 0.24 mm, and 0.36 mm, respectively. The deviations in the yaw, roll and pitch dimensions were 0.14°, 0.57°, and 0.07°. Similarly, the linear errors in the x, y, and z dimensions of the mandible were 0.2 mm,0.87 mm, and 0.83 mm, respectively. The errors in three rotational dimensions were 0.32°, 0.36°, and 0.8°. None of the linear deviations between the virtual design and the actual results were statistically significant, except for the maxillary on the y-axis (P = 0.008). The dental deviations between virtual design and actual alignment were depicted in Table 2. All standard deviations (SD) and root mean square deviations (RMSD) were less than 2 mm, except for Sample 2, which had an SD of 2.22 mm and an RMSD of 2.23 mm for the lower dentition.

**Table 1:** Statistic description of skeletal linear deviation and angular deviation

Dimension		Segment	Median	IQR	RMSD	P value
Linear deviation (mm)	x axis	Maxillary	0.17	0.07–0.27	0.21	0.910
		Mandible	0.20	0.11–0.30	0.21	0.061
	y axis	Maxillary	0.24	0.17–0.53	0.38	0.008*
		Mandible	0.87	0.13–1.23	0.94	0.394
	z axis	Maxillary	0.36	0.21–0.73	0.52	0.140
		Mandible	0.83	0.39-1.00	1.01	0.460
Angular deviation (°)	yaw	Maxillary	0.14	0.04–0.17	0.13	
		Mandible	0.32	0.12–0.36	0.28	
	roll	Maxillary	0.57	0.13–0.63	0.48	
		Mandible	0.36	0.19–0.50	0.38	
	pitch	Maxillary	0.07	0.04–0.89	0.58	
		Mandible	0.80	0.29–1.13	0.82	

IQR, interquartile range

RMSD, root mean square deviation

*P < 0.05, statistically significant difference

**Table 2:** Statistic description of simulation deviation on upper dentition and lower dentition

Sample	SD			RMSD	
	Upper dentition	Lower dentition		Upper dentition	Lower dentition
1	1.37	1.54		1.73	1.56
2	1.40	2.22		1.42	2.23
3	1.14	1.64		1.15	1.75
4	1.56	1.28		1.57	1.28
5	1.85	1.21		1.85	1.22

SD, standard deviation

## Discussion

The combination of orthognathic surgery and orthodontics is a requisite approach for dento-maxillofacial deformities correction, inspiringly motivated by advanced digital technology [3, 4, 17]. With precise diagnosis and treatment arranging, digital therapy is the future trend [18, 19]. To a large extent, computer-assisted surgery (CAS) facilitates orthognathic surgery alteration, which includes 3D reconstruction, virtual surgical planning and simulation, computer-aided manufacturing (CAD/CAM), and so on [20]. Based on virtual reality or augmented reality, virtual surgery can realize not only objective and quantitative development of the surgical plan, but also good communication among the treatment team to formulate integrated and systematic procedures. Noteworthily, the introduction of Invisalign extends the digital simulating phase forward and backward to the entire treatment procedure by virtual design on teeth movement.

Conventional fixed appliance orthodontics is widely acknowledged to perform well on combined orthodontic-orthognathic treatment [21, 22]. The clear aligner was previously deemed to correct simple malocclusions, such as mild crowding or non-extraction cases. Furthermore, some studies have verified that clear aligners can achieve satisfying results as conventional orthodontics and have some intrinsic advantages. Zhang et al. pointed out that clear aligners can better meet the aesthetic demands of adult patients with fewer dietary restrictions and easier to maintain oral hygiene and periodontal health [13]. Ke et al. investigated that Invisalign orthodontic aligners were equally effective in correcting malocclusions and even offered advantages over conventional orthodontics in terms of segmented tooth movement and reduced treatment periods [7]. Kankam et al. demonstrated that complex multiple-jaw orthognathic procedures were performed successfully with Invisalign orthodontic aligners without compromise on perioperative and short-term clinical outcomes [23]. Reliable dental alignment and preservation of periodontal tissue promote clear aligners’ popularity as an alternative, particularly in adult patients. However, simply as an orthodontic technique, the Invisalign system can not utilize digital features entirely in the joint treatment procedure; thus, three components of combination therapy remain separated and are not systematically integrated.

The sequential and methodic approach proposed in this study initially explored fully digital management for skeletal Class III malocclusion, which focused on incorporating the entire orthodontic process before and after the surgery into the digital simulating process so that the postoperative orthodontics were continued in accordance with the preoperative orthodontic design and avoided postoperative rescanning of the dentition. Moreover, preliminary accuracy evaluation of the fully digital process got satisfactory outcomes. Except for the linear deviation in the maxillary on the y-axis, all the linear deviations in the skeletal anatomy were less than 1 mm, and none of the differences between the virtual design and the actual results were statistically significant. This exceptive deviation on the y-axis could be due to a proclivity for anterior-posterior recurrence of the maxillary segment in patients with skeletal Class III malocclusion, particularly those with the excessively depressed paranasal region and significant surgical anterior movement. All of the angular deviations in yaw, roll, and pitch were less than 1°, which demonstrates the high accuracy of the virtual surgical simulation on rotation. The simulation deviation of dental alignment was less than 2 mm in most cases, only one case was more than 2 mm. In contrast to the precise movement of the bony structures during virtual design and the impact of the spatial position of the bony structures on the external appearance, minor deviations during the orthodontic process have no effect on the final occlusion. Because clear aligners are made of elastic material, even if the design differs slightly from the existing alignment, the aligners can also be worn.

Compared to conventional joint orthodontic-surgical treatment, this novel approach has several advantages, with non-inferior clinical outcomes and better aesthetic performance [23]. Firstly, the digital simulation feature of the Invisalign system outperforms fixed appliance orthodontics in terms of visualization and predictability of the long-term treatment process. At the same time, the clear aligner is more favorable for periodontal health and oral hygiene maintenance. Secondly, the incorporation of the Invisalign system ultimately connects digital components of the procedure and realizes complete digitalization with more convenient communication between sections. Furthermore, orthodontic and surgical simulation can visualize both the post-treatment occlusal relationship and skeletal location early in the procedure, which facilitates informing and explaining treatment plans to patients and receiving timely feedback. At the same time, it has some disadvantages and some inadequacies that need to be improved. The fully digital approach necessitates professional and practiced collaboration; but some computer-aided surgery equipment and experienced doctors are required, which may hinder its widespread promotion. Invisalign is effective in the treatment of mild to moderate malocclusion, but it falls short of conventional orthodontics in terms of achieving adequate occlusal contact and closing extraction spaces [24–26]. Due to each aligner being worn 20–22 h a day and changed frequently, patient compliance is highly demanded. At the same time, this study was a preliminary exploration, only small group patients with skeletal class III patients were enrolled. Therefore, large number of patients with different types of deformities need to be investigated in future.

Nonetheless, with the improvement of digital technology and materials of clear aligners, the fully digital approach will be a promising alternative in the treatment of dento-maxillofacial deformities, with concrete reliability of accurate simulation and a favorable connection between orthodontics and orthognathic surgery.

## Conclusions

Above all, a novel approach of fully digital treatment procedure was explored to correct skeletal Class III malocclusion in this study. All five included patients got satisfactory outcomes in both facial appearance and functional occlusion. It could be an alternative option for the treatment of dento-maxillofacial deformities with integrated digital technology and systematic procedures to raise treatment efficiency.

## Data Availability

All data generated and/or analyzed during the current study are available from the corresponding author on reasonable request.

## List of Abbreviations

BSSRO: Bilateral sagittal split ramus osteotomy
CAS: Computer assisted surgery
CAD/CAM: Computer-aided designing/Computer-aided manufacturing
OGS: Orthognathic surgery
RMSD: Root mean square deviations
SD: Standard deviations
VSP: Virtual surgical planning
